# The first Tunisian case of postorgasmic illness syndrome: A case report

**DOI:** 10.1002/ccr3.5120

**Published:** 2021-11-19

**Authors:** Ghada Hamdi, Hanen Ben Ammar, Oumaima Charaa, Lina Brahmi, Amira Maamri, Haifa Zalila

**Affiliations:** ^1^ Faculty of Medicine of Tunis Tunis El Manar University Tunis Tunisia; ^2^ Psychiatry Department Razi Hospital Manouba Tunisia

**Keywords:** post‐ejaculatory pain, postorgasmic illness syndrome, sexual avoidance, sexual dysfunction

## Abstract

Postorgasmic illness syndrome (POIS) is a rare and singular syndrome. About fifty cases have been reported in the medical literature. Through a clinical observation, we illustrate the first case diagnosed in Tunisia and the difficulties in the etiological diagnosis and the therapeutic management of this syndrome. Given the shortage of cases reported in the literature, the syndrome of POIS remains poorly identified and subsequently misdiagnosed. The clinical diagnosis is relatively simple, yet etiological and therapeutic questions remain to overcome.

## Graphical abstract

The diagnosis of postorgasmic illness syndrome is essentially clinical. Postorgasmic illness syndrome remains poorly identified and misdiagnosed. There are no recognized or codified treatment modalities for postorgasmic illness syndrome.

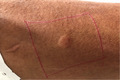

## 1 INTRODUCTION

The Postorgasmic illness syndrome (POIS) is a rare syndrome, recently described by two Dutch doctors Waldinger and Schweizer in 2002^1^ which includes a set of physical and cognitive symptoms, appearing electively after ejaculation. It can last for several days.^2^

About only 50 cases have been reported in the medical literature.^2^ The Prevalence of POIS remains unknown, likely underdiagnosed still mysterious, owing to a paucity of studies, but is most probably underreported. Symptoms occur for a few seconds to a few hours after ejaculation, whether after intercourse, masturbation, or spontaneously during sleep. These disorders reproduce almost every ejaculation.

We report a rare case of POIS that illustrates the clinical polymorphism and therapeutic difficulties of this syndrome and discusses etiopathogenic hypotheses.

## 2 CLINICAL CASE

Mr. MT, 32‐year‐old has been married to his cousin for 11 years and has 5 children. He had no somatic or psychiatric history. The patient has a secondary school level and works as a builder. He has no history of past or present substance use a side of smoking weaned for 4 years.

He was referred to the psychiatric examination at Mannouba's Razi Hospital, Tunisia, after seeing urologists, neurologists, and general practitioners. Indeed, a few minutes (10–15 mn) after each ejaculation, he felt intense fatigue, exhausted and severe muscle, bone and joint pain that persisted for a few days (3–7 days). He also reported the straights of sneezing with eye redness and sensations of itching throughout the body as well as intense impaired concentration.

The patient also reported temporary dental pain that accompanied disappeared spontaneously later.

The symptoms have been reported in all ejaculations, independently with the sexual activity “nocturnal emission, masturbation or vaginal ejaculation.”

The protests began with puberty at the age of 14 years old “his first masturbation.” The symptoms increased in severity and duration with age: at first, they lasted 3 days, which drove the patient to take days off but in the last 2 years these symptoms last for 7 days causing financial difficulties.

The evolution of these symptoms was complicated by a sexual avoidance behavior in spite of a preserved sexual desire and rigid erections. The frequency of the sexual intercourse went from 2 per week to 1 per month. Since 2 years, secondary premature ejaculation has been installed.

On examination, the patient was in good general health, well constructed, and had complete secondary sexual characteristics. His weight was 97 kg, his height was 180 cm, and his blood pressure was 120/80 mm/Hg.

Clinical examination, particularly neurological and urological, was strictly normal. No abnormalities were detected in further investigations: The results of laboratory tests routine (CBC, renal function, blood glucose) and the hormone levels (FT4, TSH, prolactin, cortisol, Testostérone, DHEA) were normal (Table 1).

**TABLE 1:** Biological tests of the patient

Biological tests	Results
DHEA	679 ng/mL (2357nM/L1)
FT4	17.78 pmol/L
TSH	1.29 μUI/mL
FSH	3.09 mUI/mL
LH	4.63 mUI/mL
Testosterone	280.5 ng/dL
Prolactin	179.1 Mui/L
Cortisol	243.3 nmol/L

**FIGURE 1:**
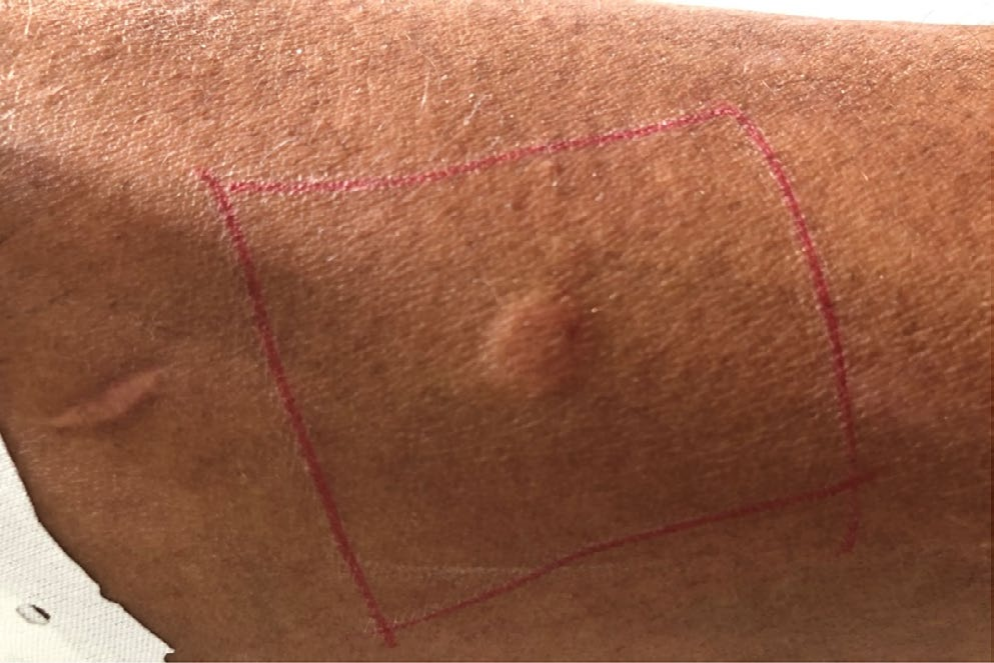
Prick positive skin test with autologous patient sperm

In order to investigate the immunoallergic origin of the POIS,^3^ the skin prick test was performed on extremely diluted sperm: The sperm freshly emitted from the patient was diluted to a concentration of 1: 40,000, then injected intracutaneously into the palmar surface of the left forearm, and compared to a cutaneous reaction to placebo with 0.9% intracutaneous saline. This test came back positive (Figure 1).

This case report was revised to comply with recommendations of the Case Report guidelines, and an informed consent publication was obtained from the patient.

## 3 DISCUSSION

We report an interesting case of POIS. The particularity of our observation consists on the rarity of the cases described in the literature, and this is to our knowledge the first reported case in Tunisia.

### 3.1 Diagnostic criteria and classification

The diagnosis of the POIS is essentially clinical. Waldinger et al submitted 5 preliminary diagnosis criteria^4^ which have recently been validated by Strashny^5^:

#### 3.1.1 Criterion I

The patient must have one or more of the following symptoms: flu‐like or extreme tiredness, muscle weakness, fever or sweating, mood and/or irritability, memory disturbances and concentration problems, incoherent speech, nasal congestion or clear nasal discharge, tingling of the eyes.

#### 3.1.2 Criterion II

All these symptoms occur immediately (within seconds), rapidly (in the minutes) or in the hours following ejaculation, whether it is caused by coitus, a masturbatory act or occurs spontaneously during sleep.

#### 3.1.3 Criterion III

These symptoms occur almost every ejaculation, that is to say in more than 90% of cases in such context.

#### 3.1.4 Criterion IV

Most of these symptoms last between 2 and 7 days.

#### 3.1.5 Criterion V

They disappear spontaneously.

These criteria were all present in our patient. The symptoms may vary from one individual to another in their nature, intensity, and duration, but they are relatively constant in the same individual.

Waldinger et al further stratify the symptoms of criterion 1 into 7 clusters^6^:

#### 3.1.6 Cluster 1 (general)

Extreme fatigue, exhaustion, palpitations, difficulty in finding words, incoherent speech, dysarthria, difficulty concentrating, rapid irritation, phonophobia, photophobia, depressed mood.

#### 3.1.7 Cluster 2 (flu‐like)

Fever, caller extreme sweating, chills, flu syndrome, feeling of being sick, feeling cold.

#### 3.1.8 Cluster 3 (head)

Headache, fog in the head, heavy feeling in the head.

#### 3.1.9 Cluster 4 (eyes)

Burning, red eyes, blurred vision, watery and irritated eyes, itchy eyes, eye pain.

#### 3.1.10 Cluster 5 (Nose)

Congested nose, runny nose, sneezing.

#### 3.1.11 Cluster 6 (throat)

Dirty taste in the mouth, dry mouth, sore throat, tickling cough, hoarse voice.

#### 3.1.12 Cluster 7 (muscles)

Muscle tension in the back or neck, muscle weakness, muscle pain, heavy legs, muscle stiffness.

There is also primary POIS that manifests from the first ejaculations during puberty or adolescence and later secondary cases.^6^ The symptomatology described by our patient started at the age of 14 years and later, it had a primary form of POIS which is the most common form.^7^

## 4 IMPACT OF POIS ON QUALITY OF LIFE

The quality of life of patients with POIS is strongly affected by the physical and psychological effects. It may have a significant effect on sufferers and their partners. Patients develop adaptive measures: they schedule sex so that symptoms, that can last a week, do not affect their work or studies. Others abstain completely. This abstinence and the overcontrol of sexual activity can contribute to a lifelong premature ejaculation but the association of POIS and premature ejaculation need to be more clarified. Fears of rejection, stigma, and non‐acceptance drive some patients to avoid engaging in lasting relationships.

### 4.1 Pathophysiology

Research on the physiopathological mechanisms of POIS is only in its infancy. The etiologies remain to be discussed and it is still not well elucidated.

The hypothesis first proposed by Waldinger et al defined POIS as an immune‐allergic phenomenon which consisted of an inflammatory reaction to a substance in the seminal fluid of man.^8^ This theory has been confirmed by a positive reaction to skin prick test (SPT) in patients with POIS at highly dilated sperm autologous.^4^ Guarding the same theory, Kim et al. objectified the presence of semen‐specific immunoglobulin EIN while investigating a patient with POIS^9^ but this high level of IgE remains to be discussed.^3^

Jiang et al, on their side, found there was no proof of semen‐specific IgE and instead postulated that chemical imbalances in the brain could be the physiological basis of POIS, generating symptoms comparable to opioid withdrawal. They have discussed endogenous u‐opioid receptor injury, suggesting that during orgasm, there is a consumption of excessive amounts of endogenous opioids, resulting in symptoms similar to opioid withdrawal.^7^

Ashby and Goldmeier concluded that it is rather done with a neuroendocrine response and disordered cytokines after improvement of symptoms under nonsteroidal anti‐inflammatory drugs.^10^

Others pathophysiological hypotheses include transient deregulation and hypersensitivity were suggested.

A recent study hypothesized that an acute compression axonopathy at the proprioceptive sensory terminals in the muscle spindle can cause POIS.^11^

## 5 MANAGEMENT

Postorgasmic illness syndrome is a rare, probably underdiagnosed, and underreported illness. To our knowledge, there are no recognized or codified treatment modalities.^12^ Patients with POIS were treated with sperm autologous hyposensitization therapy, antihistamine agents,^8^ selective serotonin reuptake inhibitors, and benzodiazepines^13^ nonsteroidal anti‐inflammatory drugs such as diclofen.^10^

Silodosin, a highly selective alpha1A‐blocker, which is literally source of ejaculation can be used as a possible symptomatic treatment of POIS.^3^

A first successful treatment of POIS using HCG to a patient with testosterone (T) deficiency argues to multiply hormonal investigations in case of POIS.^14^

At long last, various recounted adjuvant treatments have been proposed to treat POIS such as olive leaf, niacin, fenugreek, saw palmetto, and Wobenzym.

The crucial benefit of the case is to point out that this diagnosis implicates the collaboration of multiple specialties. Our patient had several investigations before retaining the diagnosis which delayed therapy.

## 6 CONCLUSION

Given the shortage of cases reported in the literature, the syndrome of postorgasmic illness remains poorly identified and subsequently under diagnosed. The clinical diagnosis is relatively simple, yet etiological and therapeutic questions remain to overcome.

## CONFLICTS OF INTERESTS

All authors declare that they have no conflicts of interest to disclose.

## AUTHOR CONTRIBUTION

GH and HBA conceived the ideas and led the writing. OC, LB, and AM involved in writing. HZ did the editing.

## ETHICAL APPROVAL

An informed consent publication was obtained from the patient.

## CONSENT

Published with the written consent of the patient.

## Data Availability

The data that support the findings of this study are available from the corresponding author upon reasonable request.
